# Do Mendelian randomization findings for circulating proteins in polycystic kidney disease replicate? A two-stage, two-platform study

**DOI:** 10.1093/ckj/sfag389

**Published:** 2026-10-29

**Authors:** Yiyi Zhang, Cheng Xue, Jiangming Zhang, Sen Zhou, Tianxi Liu, Zhiguo Mao

**Affiliations:** First Clinical Medical College of Lanzhou University, Lanzhou, China; Department of Nephrology, Shanghai Changzheng Hospital, Naval Medical University, Shanghai, China; First Clinical Medical College of Lanzhou University, Lanzhou, China; First Clinical Medical College of Lanzhou University, Lanzhou, China; First Clinical Medical College of Lanzhou University, Lanzhou, China; First Clinical Medical College of Lanzhou University, Lanzhou, China; Department of Nephrology, Shanghai Changzheng Hospital, Naval Medical University, Shanghai, China

To the Editor,

Mendelian randomization (MR) is increasingly used to nominate circulating proteins and drug targets for polycystic kidney disease (PKD), such as sex hormone-binding globulin (SHBG) [1] and 3-hydroxy-3-methylglutaryl-coenzyme A reductase (HMGCR), proxied by low-density lipoprotein (LDL) cholesterol-associated variants in its gene region [2]. Both studies used outcomes with fewer than 1000 cases, reported mostly nominal associations and had not been tested in a larger independent cohort. We asked whether such findings replicate.

We first applied the common single-dataset design, testing SOMAscan *cis-*acting protein quantitative trait loci (*cis-*pQTLs) from INTERVAL against PKD in combined BioBank Japan and UK Biobank (BBJ + UKB) data (GWAS Catalog GCST90018904; 934 cases). Of 502 proteins, 22 were nominally significant (about 25 expected by chance), leaving 19 candidates after Steiger filtering. We then tested these candidates (clumped *cis-*pQTLs) and the wider proteome (strongest *cis-*pQTL per assay) with deCODE SomaScan v4 [3] and UKB Olink Explore 3072 [4] (2416 platform-specific assays) in FinnGen R13 cystic kidney disease (ICD-10 Q61; 1176 cases) [5]. The FinnGen outcome shared no samples with the screening outcome or either exposure, whereas the UKB Olink exposure overlapped with the UKB component of the screening outcome. We used Wald ratios or inverse-variance weighting, false discovery rate (FDR) control within each platform, and coronary heart disease as a positive control.

In 18 of 30 platform-specific tests, the 95% confidence interval (CI) excluded the screening estimate (Fig. 1A). Only GP1BA on Olink met its prespecified validation criterion (same direction, *P* < .05), but the signal was driven by a variant nearly independent of the screening instrument (*r*^2^ = 0.005) and was not reproduced in BBJ + UKB. The previously reported SHBG association [1] was not significant on either platform (odds ratio [OR] 1.05, 95% CI 0.80–1.38, Olink; OR 1.15, 95% CI 0.70–1.90, SomaScan), although exposure definitions and instruments differed. The previously reported association for LDL cholesterol proxied by variants in the HMGCR region (OR 3.05, 95% CI 1.19–7.84) [2] was not significant in FinnGen R13 (OR 1.78, 95% CI 0.63–5.00); however, the outcome samples partly overlapped and the original study did not specify the OR unit. Proteome-wide, no protein reached FDR < 0.05 (minimum 0.71 for SomaScan and 0.74 for Olink; Fig. 1B), and nominal FinnGen signals replicated in BBJ + UKB at chance rates (0/67 SomaScan, 2/71 Olink). For coronary heart disease, the pipeline identified 19 FDR-significant proteins per platform, including PCSK9, LPA, and IL6R, showing that it detects strong signals when adequately powered.

**Figure 1:**
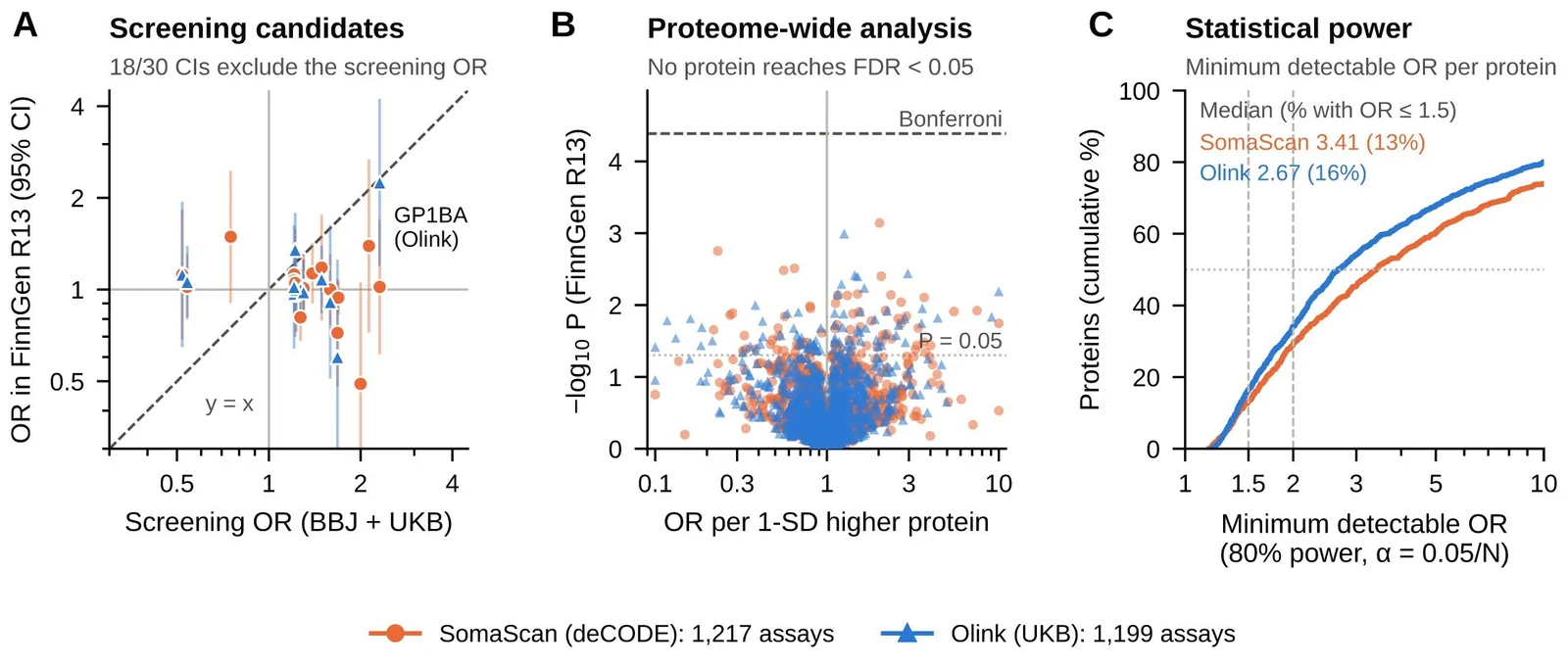
Re-tests of screening candidates, proteome-wide analysis, and statistical power in FinnGen R13 cystic kidney disease. (A) Screening OR (SOMAscan *cis-*pQTLs against BBJ + UKB) versus OR in FinnGen R13 for the 30 platform-specific tests of the 19 screening candidates; vertical lines show 95% CIs of the FinnGen estimates (truncated at the axis limits), the dashed line is y = x, and GP1BA on Olink is labelled. (B) Volcano plot of the proteome-wide analysis, with OR per 1 SD higher protein level on a log scale; the dashed line is the Bonferroni threshold (0.05/1217, the more stringent of the two platforms), the dotted line is *P* = .05, and estimates outside the axis range are drawn at its edge. (C) Cumulative distribution of the minimum detectable OR (80% power, two-sided α = 0.05/*N*, where *N* is the number of proteins tested on each platform); dashed lines mark OR = 1.5 and 2, and the dotted line at 50% marks the median. Circles, SomaScan v4 (deCODE); triangles, Olink Explore 3072 (UKB); the key below the figure identifies the two platforms in all panels. BBJ, BioBank Japan; CI, confidence interval; *cis-*pQTL, *cis-*acting protein quantitative trait locus; FDR, false discovery rate; OR, odds ratio; SD, standard deviation; UKB, UK Biobank.

Limited power is an important explanation. At the Bonferroni threshold, the median minimum detectable OR was 3.41 (SomaScan) and 2.67 (Olink), and only 13% and 16% of proteins had at least 80% power to detect an OR of 1.5 (Fig. 1C); about 6900–10 800 cases would be needed for half of the proteins to reach this power. Nominal findings are therefore prone to false positives and inflated effect estimates.

*Post hoc*, only one screening instrument, the H6PD missense variant p.Asp151Ala, was directionally consistent in FinnGen and passed Bonferroni correction (per-allele OR 1.28, 95% CI 1.14–1.44; *P* = 2.1 × 10^−5^), short of genome-wide significance; the evidence did not support mediation through plasma H6PD levels, and functional studies could test whether altered H6PD activity, rather than circulating abundance, underlies the association.

Lack of replication is not refutation; smaller effects and effects on disease progression remain untested. At current sample sizes, however, single-dataset protein MR findings in PKD should not by themselves guide drug-target development. We recommend reporting power or minimum detectable effects, validating findings in cohorts without sample overlap after verifying outcome composition, applying multiple-testing correction within each platform, and including a positive control.

## Supplementary Material

sfag389_Supplemental_File

## Data Availability

All analyses used publicly available summary statistics: INTERVAL SOMAscan pQTLs (Sun *et al., Nature* 2018), deCODE SomaScan v4 [3], UK Biobank Olink [4], FinnGen R13 [5], the BBJ + UKB PKD GWAS (Sakaue *et al., Nature Genetics* 2021; GWAS Catalog GCST90018904), and Global Lipids Genetics Consortium data (Willer *et al., Nature Genetics* 2013). All results generated in this study are provided in Supplementary Tables S1–S15. The analysis code is available from the corresponding author on reasonable request.

## References

[1] Zhang C, Yang Y. 35 blood and urine biomarkers in polycystic kidney disease: a bidirectional Mendelian randomization study. Biotechnol Biotechnol Equip. 2024;39:2435385. 10.1080/13102818.2024.2435385

[2] Lian Z, Liang Z, Chen Q et al. Association between lipid-lowering drug targets and the risk of cystic kidney disease: a drug-target Mendelian randomization analysis. Ren Fail. 2025;47:2491657. 10.1080/0886022X.2025.2491657 PMC12035922 40289090

[3] Ferkingstad E, Sulem P, Atlason BA et al. Large-scale integration of the plasma proteome with genetics and disease. Nat Genet. 2021;53:1712–21. 10.1038/s41588-021-00978-w 34857953

[4] Eldjarn GH, Ferkingstad E, Lund SH et al. Large-scale plasma proteomics comparisons through genetics and disease associations. Nature. 2023;622:348–58. 10.1038/s41586-023-06563-x PMC10567571 37794188

[5] Kurki MI, Karjalainen J, Palta P et al. FinnGen provides genetic insights from a well-phenotyped isolated population. Nature. 2023;613:508–18. 10.1038/s41586-022-05473-8 PMC9849126 36653562

